# Experiences of pediatric physiotherapists on the use of walking orthoses in children with cerebral palsy: A quantitative examination

**DOI:** 10.1097/MD.0000000000044835

**Published:** 2025-10-03

**Authors:** Halil Hakan Uysal, Cemile Bozdemir Ozel, Sinem Asena Sel, Hande Fidan, Eda Burc, Sabri Erdem, Mintaze Kerem Gunel

**Affiliations:** a Department of Prosthetics and Orthotics, Vocational School of Health Services, Eskisehir Osmangazi University, Eskisehir, Turkey; b Department of Physiotherapy and Rehabilitation, Faculty of Health Sciences, Eskisehir Osmangazi University, Eskisehir, Turkey; c Department of Physiotherapy and Rehabilitation, Faculty of Health Sciences, Antalya Bilim University, Antalya, Turkey; d Department of Physiotherapy and Rehabilitation, Faculty of Health Sciences, Istanbul Kent University, Istanbul, Turkey; e Faculty of Physiotherapy and Rehabilitation, Hacettepe University, Ankara, Turkey; f Faculty of Business, Dokuz Eylül University, Izmir, Turkey.

**Keywords:** cerebral palsy, ICF, mobilization, orthoses

## Abstract

Orthoses play an important therapeutic role in the rehabilitation of children with cerebral palsy (CP). Studies examining the factors that physiotherapists consider when prescribing orthotics are limited in the literature. This study aimed to examine physiotherapists’ perspectives and experiences with using walking orthoses in patients with CP. A total of 207 physiotherapists working with children with CP were included in this online cross-sectional study. The questionnaire consisted of examining the knowledge of the physiotherapist about walking orthoses, such as the types of orthoses that are preferred in clinical decision-making, which evaluation methods are used in the decision-making process in orthosis selection, and changes in orthosis usage based on International Classification of Functioning. Less than half of physiotherapists rated their knowledge of orthotics as “good” (n = 102, 49.3%). The most commonly used clinical methods to recommend orthoses are gait pattern, muscle tone, observational gait analysis, and activity targets. Physiotherapists reported a decrease in the severity of the structural disorder in the legs and feet and improvement in participation in daily life and activities after using orthoses. In contrast, social policies and health services pose a major barrier to children’s use of orthoses. According to the International Classification of Functioning Framework analysis, mobilization was related to activities of daily living, family and technology use, movement functions, social support and health, and sleep-mood-pain (*P* < .001). The study’s findings show that deformity in the lower extremities, mobilization, and activities of daily living in children with CP improved after the use of orthoses. Increasing physiotherapists’ knowledge of orthoses may be more advantageous in terms of the effectiveness of the treatment applied.

## 1. Introduction

Cerebral palsy (CP) is a condition characterized by a disorder in movement development and posture as a result of nonprogressive damage to the developing brain. Motor disorders are often accompanied by sensory, cognitive, communication, perception, behavioral disorders, and epilepsy.^[1]^ Muscle tone, tendon reflexes, selective motor control, range of motion, and muscle strength are affected to varying degrees according to their effect on the central nervous system.^[2]^ The purpose of healthcare for children diagnosed with CP is to prevent deformities, reduce pain, and ensure activity and participation by applying cognitive and/or behavioral strategies.^[3]^

Orthoses play an important therapeutic role in the rehabilitation of children with CP.^[4]^ The purpose of orthotics is to increase motor function, improve gait quality, prevent contractures and deformities, and provide normal body alignment in individuals diagnosed with CP.^[5]^ The gross motor function level influences the selection of orthoses.^[6,7]^ Considering children who are independent or mobilized with a walker, the selection of orthosis aims to ensure effective gait and prevent deformities.^[5]^ In children mobilized with a wheelchair, the aim is to prevent spine and hip deformities due to increased exposure and to provide sitting function to ensure functional and social participation.^[5]^ The opinions of the family, child, orthopedist, and physiotherapist are important in the decision-making process regarding the orthosis to be used by children with CP.^[5]^

Physiotherapists play a key role in the decision-making process regarding orthoses.^[8]^ Clinicians have many different types of orthotics when recommending walking orthoses and when making decisions.^[8]^ They also preferred different evaluation methods. There are also differences of view among therapists regarding how long the orthosis is used.^[8,9]^ Clinicians have to consider the type of CP and gait pattern as well as environmental and personal factors when recommending and designing suitable orthotics. In a qualitative study conducted by Kane et al, semi-structured questions were asked to physiotherapists about the decision-making phase of walking orthoses. As a result, communication between the physiotherapist and orthotist and measuring the outcome of the proposed orthosis were determined as the basic procedures in clinical problem-solving.^[8]^ However, there is no standard for using these procedures in an integrated manner in the orthotic care of children with CP.^[8]^ There are guidelines for prescribing orthotics to children with CP.^[7–9]^ An algorithm was also created to recommend an appropriate orthotic.^[10]^ The orthosis given according to current guidelines should be targeted and determined within the framework of the International Classification of Functioning, Disability, and Health–Child and Youth Version (ICF-CY) in line with body structure and functions, activity, participation, and environmental and child-specific factors.^[11]^ But these guidelines are lacking. In Turkey, the effect of walking orthoses on structure, function, activity, and participation has been examined from the perspective of ICF-CY.^[8–13]^ However, there only a limited range of studies have examined the factors that pediatric physiotherapists consider when choosing orthoses as well as the perspectives and experiences of physiotherapists in relation to orthosis recommendations.

Overall, the current study aimed to examine the perspectives and experiences of physiotherapists regarding the use of walking orthoses in patients with CP. The aim of this study was to acquire the knowledge of physiotherapists working in the field of pediatric rehabilitation for walking orthoses and to develop materials and algorithms that can help physiotherapists working in the field of pediatric rehabilitation in recommending walking orthoses clinically and academically in the future.

## 2. Methods

### 2.1. Participants

The study population consisted of pediatric physiotherapists with at least 1 year of experience. Physiotherapists who were not working in pediatric physiotherapy and rehabilitation and who refused to participate were excluded from the study. The sample size was calculated a priori as 164 using the formula for cross-sectional studies, with a margin of error set at 5% and confidence levels at 80%. Physiotherapists were contacted via e-mail or social media and invited to participate in the study. In addition, the study was announced by the Turkish Physiotherapy Association and social media accounts of the researchers involved in the study, and a questionnaire form created through Google Forms was sent to physiotherapists who accepted the study. This study was conducted between December 2021 and October 2022 with ethical approval from the Non-Interventional Clinical Research Ethics Committee (Number: GO 21/1259 Decision No: 2021/19-07) of Hacettepe University.

### 2.2. Assessment

The participants were given a questionnaire to fill out on various social media platforms such as WhatsApp and Gmail. All participants provided informed consent for the survey questionnaire. Informed consent was obtained from the participants to complete the questionnaire, and they agreed to participate in the research. Informed consent was obtained during the initial contact with the physiotherapists. The questionnaire consisted of, 1st, consent to participate in the study; the participants had to check a box to take the questionnaire in which they confirmed that they had read and understood the procedure and agreed to participate voluntarily. All collected data were anonymous, and confidentiality of the ID details and study data was maintained at all times. Once the participant completed the questionnaire, the data were uploaded electronically. Only study investigators had access to identifiable data.

A sociodemographic information form section was used to collect information about variables, such as demographic characteristics of individuals (gender, age, height, body weight, etc), educational status, and employment status. Next, questions were asked to review the knowledge of the physiotherapist regarding walking orthoses. This section included questions such as the types of orthoses that are preferred in clinical decision-making, which evaluation methods are used in the decision-making process in orthosis selection, which orthotic material is preferred, and the recommended daily use (in hours) of the orthosis.

The questionnaires were then transferred to a digital platform. The online form was sent to physiotherapists who worked with children with CP for at least 1 year and could be contacted by investigators via their social media accounts and electronic mail addresses.

### 2.3. Statistical analysis

Statistical analyses were conducted using the IBM SPSS software (version 26.0; IBM Corp., Armonk). The demographic and clinical characteristics of the physiotherapists and International Classification of Functioning (ICF)-Core set questions were described using mean (standard deviation) or median (minimum-maximum) for the numerical variables and frequency (%) for categorical variables. IBM Statistical Package for the Social Sciences (SPSS) 23 and Analysis of Moment Structure (Amos) 24 Software (Amos Development Corporation 3000 Village Run Road Unit 103, #315 Wexford) were used for modeling. First, explanatory factor analysis was performed to determine the number of factors and factor loadings of the items. Subsequently, a structural equation model (SEM) was created according to the determined number of factors.

The relationships between activity and participation, environmental factors, and body function were examined using the SEM. Chi-square/degrees of freedom (CMIN/df; 0 < CMIN/df < 3 as good compliance), The root mean square error of approximation (RMSEA; 0 ≤ RMSEA ≤ 0.05 as good, 0.05 ≤ RMSEA ≤ 0.08 as acceptable compliance) and goodness of fit index (GFI; 0.90 ≤ GFI ≤ 1 as good compliance, 0.85 ≤ GFI ≤ 0.90 as acceptable compliance), the comparative fit index (CFI; 0.90 ≤ CFI ≤ 1 as good compliance, 0.85 ≤ CFI ≤ 0.90 as acceptable compliance) were used for model fit.^[14]^

## 3. Results

In this study, 252 physiotherapists aged 22 to 67 living in Turkey were contacted. The questionnaires were sent to 200 participants by e-mail and to the remaining 52 by telephone. However, 45 people were excluded from the study because of lack of feedback or data. As a result, 207 physiotherapists (mean age, 35 years 3 months [9 years 2 months], range 22–67 years) were included in the study. More than half of the participants were female (n = 136, 65.7%) and had an undergraduate level of education (n = 107, 51.7%). While the participants worked as physiotherapists for an average of 13 years, they also worked with children with CP for an average of 10 years in the field of pediatric rehabilitation. Most physiotherapists worked in special education centers (n = 111, 53.6%). The demographic characteristics of the physiotherapists are shown in Table 1. Although the majority of physiotherapists had not received any postgraduate training in orthosis (n = 162, 78.3%), almost all recommended lower-extremity orthoses in children with CP (n = 205, 99%). Less than half of the physiotherapists rated their knowledge of orthotics as “good” (n = 102, 49.3%), while just more than half of them felt “good” confident (n = 110, 53.1%) in recommending orthotics. More than half of the physiotherapists suggested lower-extremity orthosis to support walking and standing, protect joints, control spasticity, and support balance and therapy programs.

**Table 1:** Demographic data of physiotherapists working with children with cerebral palsy.

Physiotherapists		n (%)
Sex		
Female		136 (65.7%)
Male		71 (34.3%)
Age	X (SD)	min–max
	35.34 ± 9.21	22–67
Education level		n (%)
Bachelors		107 (51.7%)
Master degree		56 (271%)
Doctor of Philosophy		44 (21.3%)
How many years have you been working as a physiotherapist?	X (SD)	min–max
	13.29 ± 9.47	1–45
How many years have you been working in the field of pediatric rehabilitation?	X (SD)	min–max
	10.35 ± 7.99	1–34
How many years have you been working with children with Cerebral Palsy?	X (SD)	min–max
	10.08 ± 7.86	0–34
In which institutions do you work?		n (%)
Public Hospitals		30 (14.5%)
Special Education Centers		111 (53.6%)
Private Hospitals		17 (8.2%)
University Hospitals/Research and Application Centers		41 (19.8%)
Counseling Centers		18 (8.7%)
Public Institutions		5 (2.4%)
Other		2 (1%)

The participants preferred static ankle foot orthosis (AFO), supramalleolar, dynamic AFO, articulated AFO, and knee AFO in proposing lower extremity orthosis. According to physiotherapists, the most essential factors for lower-extremity orthosis were standing, muscle tone, joint protection, and joint alignment. The most commonly used clinical methods to recommend orthoses are gait pattern, muscle tone, observational gait analysis, and activity targets. In addition to the majority of physiotherapists participating in the orthosis design (n = 138, 66.7%), they preferred polyethylene as an orthotic material (n = 155, 74.9%). In addition, the most recommended view for the duration of orthosis use was 7 hours or more (n = 80, 38.6%; Table 2).

**Table 2:** Data from questions asked by physiotherapists about orthotics.

	n (%)
Did you receive any postgraduate training on orthoses in cerebral palsy?	
Yes	45 (21.7)
No	162 (78.3)
Would you recommend lower extremity orthosis for children with cerebral palsy?	
Yes	205 (99)
No	2 (1)
What is your level of knowledge about lower extremity orthoses in cerebral palsy?	
Very good	29 (14.0)
Good	102 (49.3)
Neither good nor bad	67 (32.4)
Bad	7 (3.4)
Very bad	2 (1.0)
For what purpose do you recommend lower extremity orthoses?	
After BoNT-A	141 (68.1)
After surgery	153 (73.9)
Support and improvement in walking	185 (89.4)
Supporting and improving balance	147 (71.0)
Supporting therapy program	150 (72.5)
Maintaining a joint range of	150 (72.5)
Control of spasticity	138 (66.7)
Control of muscle weakness	63 (30.4)
Protecting joints	138 (66.7)
Support and improving standing	180 (87.0)
Facilitation/maintenance hygiene	48 (23.2)
Other	7 (3.4)
How confident are you when recommending a lower extremity orthosis to a patient with cerebral palsy?	
Very good	33 (15.9)
Good	110 (53.1)
Neither good nor bad	58 (28.0)
Bad	5 (2.4)
Very bad	1 (0.5)
Which orthoses do you recommend in the treatment program?	
UCBL	165 (79.7)
Supramalleolar	190 (91.8)
Solid static AFO	192 (92.8)
Article AFO	175 (84.5)
Metal-hinged AFO	58 (28.0)
Dynamic AFO	197 (95.2)
PLS AFO	133 (64.3)
GRAFO	150 (72.5)
Anterior AFO	56 (27.1)
Reflexes AFO	106 (51.2)
Boats with AFO	116 (56.0)
KAFO	169 (81.6)
Hinged KAFO	141 (68.1)
What do you think is the most important factor when deciding whether a child with cerebral palsy needs a lower extremity orthosis?	
Joint alignment	134 (64.7)
Joint protection	127 (61.4)
Midfoot stability	95 (45.9)
Lower-extremity ROM	111 (53.6)
Muscle tone	125 (60.4)
Muscle strength	80 (38.6)
Selective motor control	86 (41.5)
Balance	108 (52.2)
Maintaining standing	136 (65.7)
Postural disorders	87 (42.0)
Walking distance	43 (20.8)
Walking speed	42 (20.3).
Gait disturbance	113 (54.6)
Other	3 (1.4)
What clinical measures do you use when recommending lower extremity orthoses?	
Evaluation of walking on video	91 (44.0)
Goniometric measurements:	86 (41.5)
Bone/joint alignment of the foot and leg	147 (71.0)
Limb length (mm)	121 (58.5)
Gait pattern/gait disturbances	183 (88.4)
Muscle tone	153 (73.9)
Muscle strength	104 (50.2)
Gross motor function	106 (51.2)
Selective motor control	79 (38.2)
Pain	57 (27.5)
Spatio-temporal gait kinematics	52 (25.1)
Observational gait analysis	156 (75.4)
3D gait analysis	27 (13.0)
Participation	92 (44.4)
Activity goals	135 (65.2)
Parental preference and satisfaction	76 (36.7)
Children’s preferences and satisfaction	95 (45.9)
Other	2 (1.0)
Which clinical measures do you use in the reassessment phase after using lower extremity orthoses?	
Evaluation of walking in video	90 (43.5)
Goniometric measurement	62 (30.0)
Bone/joint alignment of foot and leg	126 (60.9)
Limb length (mm)	80 (38.6)
Gait pattern/gait disturbances	162 (78.3)
Muscle tone	115 (55.6)
Muscle strength	75 (36.2)
Gross motor function	93 (44.9)
Selective motor control	63 (30.4)
Pain	68 (32.9)
Spatio-temporal gait kinematics	45 (21.7)
Observational gait analysis	153 (73.9)
3D gait analysis	16 (7.7)
Participation	96 (46.4)
Activity goals	126 (60.9)
Parental preference and satisfaction	84 (40.6)
Children’s Preference and Satisfaction	104 (50.2)
Other	1 (0.5)
Do you play a role in the design of lower extremity orthoses?	
Yes	138 (66.7)
No	36 (17.4)
I could not afford	33 (15.9)
Which orthotic material do you prefer most?	
Polyethylene	155 (74.9)
Polypropylene	123 (59.4)
Metal	4 (1.9)
Other	1 (0.5)
How many hours a day do you recommend using lower extremity orthoses?	
4 h and less than	42 (20.3)
5 h	19 (9.2)
6 h	63 (30.4)
7 h and up	80 (38.6)
Night,	56 (27.1)
Another	22 (10.6)

AFO = ankle foot orthosis, BoNT-A = botulinum toxins, GRAFO = ground reaction ankle foot orthosis, KAFO = knee ankle foot orthosis, PLS AFO = posterior leaf spring ankle foot orthosis, ROM = range of motion, UCBL = The University of California Biomechanics Laboratory (UCBL) Foot Orthoses.

Considering the body functions of the children after the use of orthoses, an increase in energy levels, a decrease in fatigue, an increase in joint functions, a decrease in muscle tone disorders, an increase in the control of voluntary movements, and improvements in gait, muscle, and movement functions were observed. According to physiotherapists, the severity of structural disorders in the leg and foot decreases significantly in children after using orthoses. According to physiotherapists, improvements in participation in daily life and activities such as changing body position, maintaining body position, moving objects with lower extremities, walking around different places, using equipment, caring for body parts, toileting, and dressing have been observed in children. According to physiotherapists, orthoses moderately facilitate the daily lives of children and their wanderings inside and outside the home. In addition, according to physiotherapists, the attitude of the children of the families towards the use of orthotics was moderately facilitating. By contrast, social policies and health services posed a major barrier to children’s use of orthoses (Table 3).

**Table 3:** Regression weights of factors and the relationship between factors.

	Regression weights	*P*
mobilization ← Sleep emotion pain	.179	.047
mobilization ← Movement Function	.704	***
Family and technology use ← mobilization	.525	***
daily activities ← Movement Function	.440	***
daily activities ← Sleep emotion pain	.465	***
Social support and health ← mobilization	.026	.916
Family and tecnology use ← Movement Function	.058	.675
Social support and health ← Movement Function	−.013	.916
b710 ← Movement Function	.926	***
b735 ← Movement Function	.888	***
b770 ← Movement Function	.924	***
d410 ← mobilization	.793	***
d450 ← mobilization	.985	***
d455 ← mobilization	.904	***
d465 ← mobilization	1.000	
d530 ← mobilization	1.001	***
e115 ← Family and technology use	1.000	
e120 ← Family and technology use	.087	.529
b780 ← Movement Function	1.000	
d460 ← mobilization	.893	***
d415 ← mobilization	.807	***
d435 ← mobilization	.927	***
b760 ← Movement Function	.945	***
d520 ← Daily activities	1.000	
d540 ← Daily activities	.927	***
d920 ← Daily activities	.857	***
e410 ← Family and technology use	.145	.467
e575 ← Social support and health	1.000	
e580 ← Social support and health	28.235	.916
b134 ← Sleep emotion pain	1.000	
b280 ← Sleep emotion pain	1.161	***
b152 ← Sleep emotion pain	1.093	***
b130 ← Movement Function	.736	***
b270 ← Sleep emotion pain	1.021	***

*** *P* < .05.

The factor analysis resulted in the identification of 6 factors. Under the “Mobilization” factor, the variables d410, d450, d455, d465, d530, d460, d415, and d435 were identified. Under the “Family and Technology Use” factor, variables e410, e120, and e115 were identified. Under the “Daily Activities” factor, the variables d520, d540, and d920 were identified. Under the “Movement Functions” factor, variables b130, b780, b770, b735, b710, and b760 were identified. Under the “Social Support and Health” factor, variables e580 and e575 were identified. Under the “Sleep, Emotion, and Pain” factor, variables b270, b152, b280, and b134 were identified.

**Figure 1.**
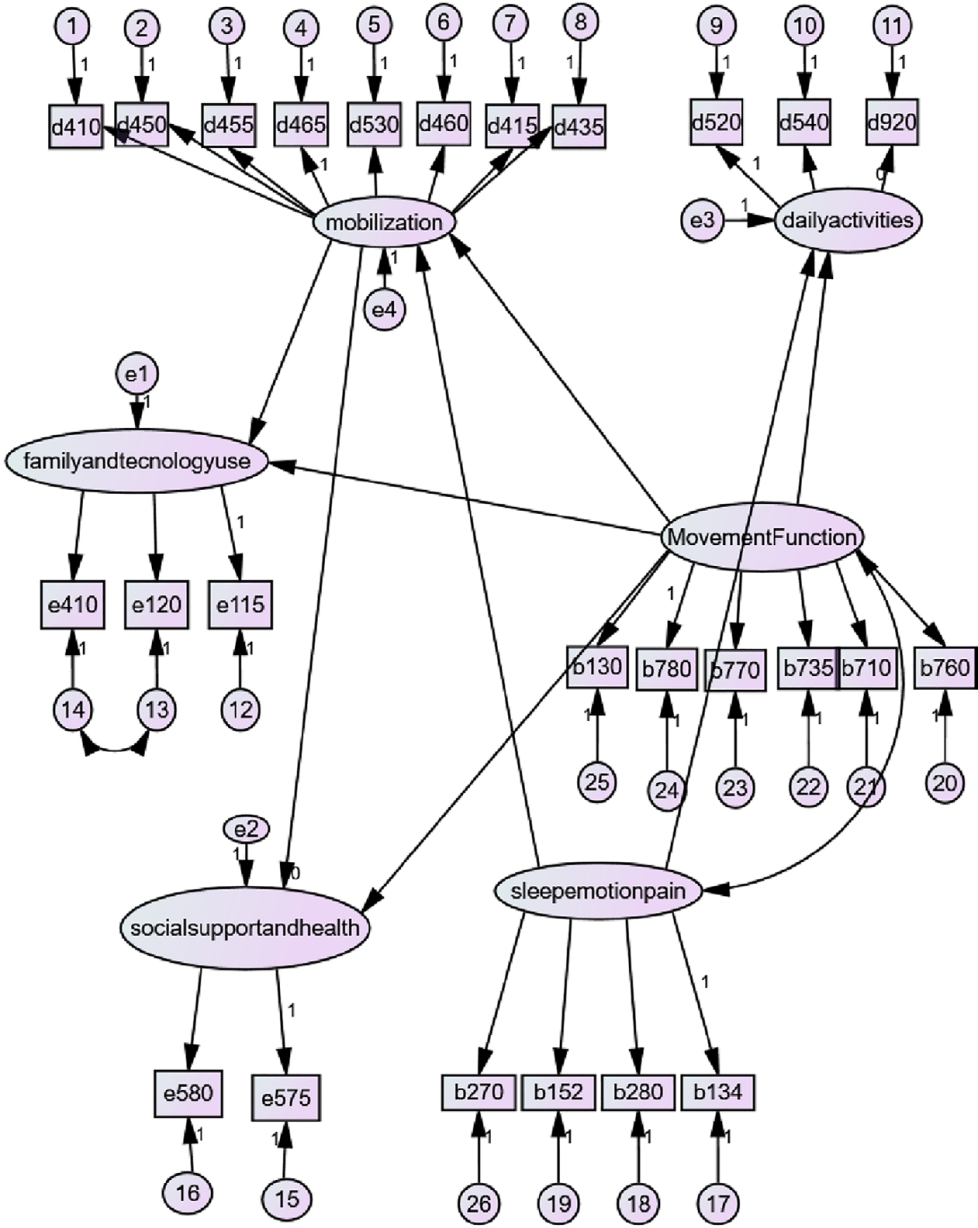
SEM model of orthosis perspective of physiotherapist. SEM = structural equation model.

The factor loads of the elements constituting the structures were compatible. We observed a significant relationship between these 6 structures (*P* < .001). According to the analysis, CMIN/df = 1.966, RMSEA = 0.68, GFI = 0.911, and CFI = 0.892. Additionally, the path coefficients, error variances, and causality between factors were also found to be significant (*P* < .05; Table 4). There was no relationship found between the factors “social support and health” and “mobilization,” family and technology use, and “Movement Function,” as well as “social support and health” and “Movement Function.” Additionally, there was no relationship found between the variables “e120” and “family and technology use,” “e410” and “family and technology use,” “e575” and “social support and health,” and “e580” and “social support and health” (Table 4). Figure 1 shows an SEM Model of the structures.

**Table 4:** Percentage responses of physiotherapists’ perspectives on the orthosis ICF framework.

ICF-codes	Orthosis perspective of physiotherapists	Percentages (%) n = 207						
		Body functions						
		No dysfunction	Mild dysfunction	Moderate dysfunction	Severe dysfunction	Complete dysfunction	Unspeficied	Not applicable
b130	Before children with cerebral palsy started to use orthoses, what level of energy level do you think the children had?	–	4.8	44.9	45.4	1.9	2.9	–
	After children with cerebral palsy start using orthoses, to what extent do you think the impairment in lower extremity energy level of children affects orthosis use?	1	16.4	48.8	30.4	0.5	2.9	–
b134	Before children with cerebral palsy started to use orthoses, how much do you think the sleep function of children was impaired?	17.9	35.7	35.7	9.2	1	0.5	–
	After children with cerebral palsy start using orthoses, to what extent do you think the sleep dysfunction of children affects the use of orthoses?	19.8	23.7	41.5	13.5	–	1	0.5
b152	Before children with cerebral palsy started to use orthoses, to what extent do you think the emotional functions of children such as sadness, happiness, anger and tension were impaired?	7.7	21.3	54.1	15.9	0.5	–	0.5
	After children with cerebral palsy start using orthoses, to what extent do you think that the use of orthosis affects the emotional functions of children such as sadness, happiness, anger and tension?	9.7	31.4	38.2	18.8	0.5	1	0.5
b270	Before children with cerebral palsy started to use orthoses, what degree of defect did the children have in the skin integrity of the orthotic area?	23.2	28	34.3	14.5	–	–	–
	After children with cerebral palsy start using orthoses, to what extent do you think the skin integrity defect in the area where the orthosis is used affects the use of orthoses?	11.1	36.7	38.2	13	–	1	–
b280	What was the level of pain do you think the children with cerebral palsy felt before they started using orthoses?	10.6	27.5	42	18.4	1	–	0.5
	To what extent do you think the use of orthoses affects the level of pain felt by children with cerebral palsy after they start using orthoses?	8.2	32.4	35.3	22.7	0.5	1	–
b455	Before children with cerebral palsy started using orthoses, what level of fatigue do you think the children felt?	2.4	14	40.6	39.6	3.4	–	–
	To what extent do you think the use of orthoses affects the level of fatigue felt by children with cerebral palsy after they start using orthoses?	2.4	29.5	38.6	28.5	–	1	–
b710	Before children with cerebral palsy started using orthoses, what degree of dysfunction do you think was seen in the mobility of their joints?	–	11.3	35.3	54.6	4.8	–	–
	After children with cerebral palsy start using orthoses, to what extent do you think the problems in the mobility of children’s joints affect the use of orthoses?	1.9	18.8	33.8	43.5	1	1	–
b735	Before children with cerebral palsy started to use orthoses, what degree of impairment do you think the children’s muscle tone was?	0.5	4.3	40.6	48.3	6.3	–	–
	After children with cerebral palsy start using orthoses, to what extent do you think the impairment in muscle tone affects the use of orthoses?	1	22.7	38.2	37.2	–	1	–
b760	Before children with cerebral palsy started using orthoses, how much impairment do you think there was in controlling their voluntary movements?	1.9	3.4	34.3	56	4.3	–	–
	After children with cerebral palsy start using orthoses, to what extent do you think the problems experienced by children while controlling their voluntary movements affect the use of orthoses?	–	20.3	39.6	38.6	0.5	1	–
b770	Before children with cerebral palsy started using orthoses, how much impairment do you think their gait was?	–	2.9	33.3	55.1	8.2	–	0.5
	To what extent do you think the use of orthoses affects the gait of children with cerebral palsy after they start using orthoses?	0.5	12.6	37.7	46.9	1	1	0.5
b780	Before children with cerebral palsy started to use orthoses, how much impairment do you think the children’s muscle and movement functions were?	–	3.4	35.7	56.5	4.3	–	–
	After children with cerebral palsy start using orthoses, to what extent do you think the impairment in muscle and movement functions of children affects the use of orthoses?	–	20.8	43	35.3	–	1	–

ICF-codes	Orthosis perspective of physiotherapists	Percentages n (%) n = 207						
		Body structure						
		No structural defect	Mild disorder	Moderate disorder	Severe disorder	Complete disorder	Unspeficied	Not applicable
s730	Before children with cerebral palsy started using orthoses, what degree of deformity did the children have in their leg and foot structures?	0.5	5.3	40.1	48.8	5.3	–	–
	After children with cerebral palsy start using orthoses, to what extent do you think the defect in the structures of the legs and feet of children affects the use of orthoses?	4.3	16.4	46.4	31.9	–	1	–

ICF-codes	Orthosis perspective of physiotherapists	Percentages n (%) n = 207						
		Activity and participation						
		No difficulty	Mild difficulty	Moderate difficulty	Severe difficulty	Complete difficulty	Notspeficied	Not applicable
d410	Before starting to use orthoses, how difficult was it for children with cerebral palsy, in your opinion, to get up from the chair and lie down on the bed, or to perform movements such as kneeling and squatting?	–	6.8	34.8	46.4	11.1	–	0.5
	In your opinion, after children with cerebral palsy start using orthoses, to what extent does the use of orthoses affect the difficulty experienced by children while performing movements such as getting up from the chair and lying on the bed or kneeling and squatting?	1.4	16.9	47.8	30.9	1.4	1	0.5
d415	How difficult was it for children with cerebral palsy to maintain kneeling, sitting and standing positions for a long time before they started using orthoses?	–	5.3	36.7	46.4	11.6	–	–
	In your opinion, after children with cerebral palsy start using orthoses, to what extent does the use of orthoses affect the strain experienced by children while maintaining kneeling, sitting and standing positions for a long time?	0.5	16.9	44.4	36.2	1	1	–
d435	Before starting to use orthoses, children with cerebral palsy had difficulty in movements such as kicking a ball or pedaling a bicycle in your opinion?	0.5	4.3	32.4	51.7	10.6	–	0.5
	In your opinion, after children with cerebral palsy start using orthoses, to what extent does the use of orthoses affect the difficulty experienced by children in movements such as kicking a ball or pedaling a bicycle?	3.9	24.2	45.9	23.7	1	1	0.5
d450	Before starting to use orthoses, children with cerebral palsy had difficulty walking short distances, long distances and on different surfaces in your opinion?	–	4.3	33.3	52.7	9.7	–	–
	In your opinion, after children with cerebral palsy start using orthoses, to what extent does the use of orthoses affect the difficulty experienced by children when walking short distances, long distances and on different surfaces?	0.5	16.4	49.3	31.4	1.4	1	–
d455	Before children with cerebral palsy started using orthotics, to what extent did they have difficulty performing movements such as running, jumping and running between obstacles, according to you?	0.5	4.3	25.1	45.4	24.6	–	–
	In your opinion, after children with cerebral palsy start using orthoses, to what extent does the use of orthoses affect the difficulty experienced by children while performing movements such as running, jumping and running between obstacles?	8.7	25.1	44.4	20.3	0.5	1	–
d460	Before starting to use orthoses, children with cerebral palsy had difficulty in moving in or out of the house according to you?	–	4.3	40.1	48.8	6.8	–	–
	In your opinion, after children with cerebral palsy start using orthoses, to what extent does the use of orthoses affect the difficulties experienced by children while moving inside or outside the home?	–	20.8	46.9	30.9	0.5	1	–
d465	How difficult was it for children with cerebral palsy to move around using a walker before they started using orthoses?	1	10.1	44	39.6	5.3	–	–
	In your opinion, after children with cerebral palsy start using orthoses, how much does the use of orthoses affect the difficulty experienced by children while walking around using a walker?	1.4	15.5	47.8	31.9	2.4	1	–
d520	How difficult was it for children with cerebral palsy to care for their body parts before they started using orthoses?	2.4	8.2	36.2	45.4	7.7	–	–
	In your opinion, after children with cerebral palsy start using orthoses, to what extent does the use of orthoses affect the difficulty children experience while caring for their body parts?	8.2	28.5	45.9	15	1.4	1	–
d530	In your opinion, how difficult was it for children with cerebral palsy to go to the toilet on their own before they started using orthoses?	0.5	4.8	34.3	49.8	10.6	–	–
	In your opinion, after children with cerebral palsy start using orthoses, to what extent does the use of orthoses affect children’s difficulty in going to the toilet on their own?	3.9	30	45.9	17.9	1.4	1	–
d540	To what extent was it difficult for children with cerebral palsy to dress before they started using orthoses?	0.5	7.7	36.7	46.9	7.7	–	0.5
	According to you, after children with cerebral palsy started using orthoses, to what extent did the use of orthoses affect their difficulty in dressing?	14.5	31.4	38.6	13.5	0.5	0.5	0.5
d920	How difficult was it for children with cerebral palsy to participate in recreational and leisure activities before they started using orthoses?	1	18.7	37.2	43	10.1	–	–
	In your opinion, after children with cerebral palsy start using orthoses, to what extent does the use of orthoses affect the difficulties experienced by children while participating in recreational and leisure activities?	3.9	29	48.8	16.9	0.5	1	–

Environmental factors								
ICF-codes – Orthosis perspective of physiotherapists							Percentages (%) n = 207	
e115 – To what extent do you think the orthosis used is facilitating or complicating the daily life of children with cerebral palsy?								
No barrier							–	
Mild barrier							13 (6.3)	
Severe barrier							16 (7.7)	
Complete barrier							3 (1.4)	
No facility							1 (0.5)	
Mild facility							27 (13.0)	
Moderate facilitator							87 (42.0)	
Substantial facilitator							58 (28.0)	
Complete facilitator							2 (1.0)	
Not applicable							–	
e120 – To what extent do you think the orthosis used is facilitating and complicating for children with cerebral palsy to move inside or outside the home?								
No barrier							–	
Mild barrier							11 (5.3)	
Severe barrier							16 (7.7)	
Complete barrier							4 (1.9)	
No facilitator							2 (1.0)	
Mild facility							31 (15.0)	
Moderate facilitator							83 (40.1)	
Substantial facilitator							57 (27.5)	
Complete facility							3 (1.4)	
Not applicable							–	
e410 – In your opinion, how facilitating or challenging is the family’s attitude towards the children with regard to the use of orthoses for your children with cerebral palsy?								
No barrier							1 (0.5)	
Mild barrier							15 (7.2)	
Severe barrier							24 (11.6)	
Complete barrier							6 (2.9)	
No facilitator							11 (5.3)	
Mild facilitator							35 (16.9)	
Moderate facilitator							48 (23.2)	
Substantial facilitator							53 (25.6)	
Complete facility							14 (6.8)	
Not applicable							–	
e575 – To what extent do you think social services and policies are facilitating or complicating the use of orthoses in children with cerebral palsy?								
No barrier							–	
Mild barrier							10 (4.8)	
Severe Barrier							40 (19.3)	
Complete barrier							58 (28.0)	
No Facilitator							25 (12.1)	
Mild facility							26 (12.6)	
Moderate facilitator							30 (14.5)	
Substantial facilitator							14 (6.8)	
Complete facility							3 (1.4)	
Not applicable							1 (0.5)	
e580 – To what extent do the health services and policies provided for children with cerebral palsy to use orthoses make it easier or more difficult for you?								
No barrier							1 (0.5)	
Mild barrier							12 (5.8)	
Severe Barrier							39 (18.8)	
Complete barrier							62 (30.0)	
No facilitator							24 (11.6)	
Mild facility							25 (12.1)	
Moderate facility							28 (13.5)	
Substantial facilitator							12 (5.8)	
Complete facility							3 (1.4)	
Not applicable							1 (0.5)	

ICF = International Classification of Functioning.

## 4. Discussion

This research was conducted to clarify the experiences and opinions of physiotherapists regarding the prescription, application, and use of walking orthoses in children with CP. In this respect, our study is similar to that conducted by Kane et al, who evaluated the criteria used by Canadian physiotherapists to create AFO prescriptions for children with CP.^[15]^ However, the critical point of this study is the changes that occur according to the ICF framework after the use of a walking orthosis in children, noticed by physiotherapists and their connections with each other.

According to the results of the study, many factors are related to mobilization, daily living activities, family and technology use, movement functions, social support and health, and sleep-mood-pain. The 1st of these headings, mobilization, was related to gross motor functions such as changing the body position, maintaining body position, moving objects with the lower extremities, moving in different places, walking, moving around, and using equipment. Öztürk et al evaluated the effects of foot and ankle orthoses and reported an improvement in walking ability and functional performance with the use of orthoses.^[16]^ The use of orthoses may have a positive effect on activities of daily living by increasing mobilization in children. Daily living activities have been found to be related to parameters regarding activity and participation, such as caring for body parts, dressing, leisure, and entertainment, in a study conducted by Naslund et al Families of children using orthoses stated that their children were more independent of daily activities and leisure time.^[17]^ It can be said that orthoses play an important role in increasing independence in daily living activities by contributing to functional development.

In the category of body functions, sub-parameters such as energy, muscle and movement, gait, muscle tone, joint mobility, and voluntary movements were shown in relation to each under the title of movement functions. Many improvements in body function have been highlighted in the literature compared to other studies conducted on children using orthoses.^[18–20]^ Although the key points in ICF-CY are activity and participation, it should not be forgotten that body functions are also significant building blocks. In addition, as in Karabulut study, sleep–emotion–pain, which is a factor affecting spasticity, is also an important determinant of daily life activities.^[21]^

Finally, when environmental factors were examined, it was noticed that health services and policies, ease of use in daily life and in different places, and family support were the determinant factors for orthosis use. All these factors can either facilitate or complicate obstacles in a child’s life. However, developing health services and policies, adapting technological products, and providing family education will be big steps in increasing children’s participation in life.

Our study is the 1st to evaluate orthosis selection according to ICF and analyze the factors affecting it. However, our study has some limitations. Firstly, physiotherapists with at least 1 year of pediatric rehabilitation experience were included in the study. While it is believed that physiotherapists in this category may possess more current knowledge, this restricts the applicability of the results to physiotherapists who have less experience or come from varied clinical backgrounds. Secondly, the subjective evaluation of the perspectives of a limited number of physiotherapists within the framework of the ICF limits the representativeness of the study’s findings to all Turkish physiotherapists at the national level.

## 5. Conclusion

This study shows what physiotherapists consider and evaluate when recommending orthoses and what may change in children after orthosis use. Considering the prevalence of orthosis use in children with CP, providing training to physiotherapists about orthoses would be more advantageous in terms of increasing the effectiveness of applied therapy. In line with the findings of this study, there is a need for future studies that evaluate the effect of orthotic use in the ICF perspective with more objective methods.

## Author contributions

**Conceptualization:** Halil Hakan Uysal, Cemile Bozdemir Ozel, Sinem Asena Sel, Eda Burc, Mintaze Kerem Gunel.

**Data curation:** Halil Hakan Uysal, Hande Fidan, Eda Burc, Mintaze Kerem Gunel.

**Formal analysis:** Halil Hakan Uysal, Cemile Bozdemir Ozel, Hande Fidan, Eda Burc, Sabri Erdem, Mintaze Kerem Gunel.

**Funding acquisition:** Cemile Bozdemir Ozel, Mintaze Kerem Gunel.

**Investigation:** Halil Hakan Uysal, Sinem Asena Sel, Hande Fidan, Mintaze Kerem Gunel.

**Methodology:** Halil Hakan Uysal, Cemile Bozdemir Ozel, Sinem Asena Sel, Mintaze Kerem Gunel.

**Project administration:** Halil Hakan Uysal, Sabri Erdem, Mintaze Kerem Gunel.

**Resources:** Halil Hakan Uysal, Cemile Bozdemir Ozel.

**Software:** Halil Hakan Uysal.

**Supervision:** Halil Hakan Uysal.

**Validation:** Halil Hakan Uysal, Sabri Erdem, Mintaze Kerem Gunel.

**Visualization:** Halil Hakan Uysal, Hande Fidan, Mintaze Kerem Gunel.

**Writing – original draft:** Halil Hakan Uysal, Sabri Erdem, Mintaze Kerem Gunel.

**Writing – review & editing:** Halil Hakan Uysal, Sinem Asena Sel, Mintaze Kerem Gunel.
